# Same-day ART initiation, loss to follow-up and viral load suppression among people living with HIV in low- and middle-income countries: systematic review and meta-analysis

**DOI:** 10.11604/pamj.2023.46.92.40848

**Published:** 2023-11-27

**Authors:** Hafidha Mhando Bakari, Oluwafemi Alo, Mariam Salim Mbwana, Swalehe Mustafa Salim, Emilie Ludeman, Taylor Lascko, Habib Omari Ramadhani

**Affiliations:** 1 President’s Office Regional Administration and Local Government, Ajira Yako, Tanzania,; 2 Center for International Health Education and Biosecurity, Maryland Global Initiatives Corporation affiliate of the University of Maryland Baltimore, Abuja, Nigeria,; 3 Primary Health Care Institute, Iringa, Tanzania,; 4 Canada Youth Group, Iringa, Tanzania,; 5 Health Services and Human Services Library, University of Maryland Baltimore, Baltimore, United States,; 6 Center for International Health, Education, and Biosecurity, University of Maryland School of Medicine, Baltimore, Maryland, United States,; 7 Institute of Human Virology, University of Maryland, School of Medicine, Baltimore, Maryland, United States

**Keywords:** Same-day ART initiation, loss to follow-up, viral load suppression, people living with HIV, low- and mid-income countries

## Abstract

**Introduction:**

in 2015, the World Health Organization recommended early antiretroviral therapy (ART) initiation after HIV diagnosis. Mixed results on the effect of same-day ART initiation (SDI) over non-same-day ART initiation (NSDI) on loss to follow-up (LTFU) and viral load suppression (VLS) necessitate further evaluation.

**Methods:**

this was a systematic review and meta-analysis of people living with HIV in low- and middle-income countries (LMICs). Multiple databases were searched from January 2016 to December 2022. VLS was defined as HIV RNA <1,000 or <400 cells/ml, depending on the study. Forest plots were used to present the pooled prevalence and 95% confidence intervals (CIs). Heterogeneity was tested by an I^2^ statistic and a p-value of <0.05 indicated its presence. Analyses were performed in STATA.

**Results:**

sixteen studies (5 clinical trials, 10 cohorts, and 1 cross-sectional) were included in the final analysis. Nine studies with 157,633 people living with HIV were analyzed for LTFU and the pooled prevalence of LTFU was 22.0% (95%CI; 18.5-25.7). The pooled prevalence of VLS was 72.7% (95%CI; 65.4-79.5%). The I^2^ statistic had a Q value of 200.62 (p<0.001) and 44.63 (p<0.001) for pooled prevalence of LTFU and VLS, respectively. Overall, compared to those who received NSDI, SDI had a significantly increased risk of LTFU (risk difference (RD)=0.04; 95%CI: 0.01-0.07). Although observational studies showed an increased risk of LTFU among SDI compared to NSDI (RD=0.05, 95%CI: 0.02-0.08), clinical trials did not. There was no statistically significant difference in VLS comparing those who received SDI vs NSDI (RD= 0.02, 95%CI: -0.03 - 0.07).

**Conclusion:**

nearly two in ten people living with HIV in LMICs who initiated ART were LTFU. SDI was associated with increased risk of LTFU. Efforts to prevent LTFU among those who receive SDI are critical to maximize its potential benefits.

## Introduction

In 2015, the World Health Organization (WHO) recommended early antiretroviral therapy (ART) initiation after HIV diagnosis [1]. It is undoubtedly that early ART initiation reduces the time to viral load suppression and subsequently HIV transmission [2-4]. Despite these benefits, delays in ART initiation exist [5-7].

Historically, in low- and middle-income countries (LMICs), persons diagnosed with HIV infection were initiated on treatment based on clinical and immunologic criteria [8]. During this pre-treatment period, these people received counselling on HIV infection and the benefits of ART. A study in South Africa showed up to 15% of people deferred treatment up to six months after HIV diagnosis and those who were not socially prepared and mobile populations were more likely to defer treatment [9]. Prior studies documented the benefits of these counselling sessions, including getting the time to understand and accept HIV diagnosis [10-12]. There have been concerns of whether early ART initiation, preferably on the same day of HIV diagnosis, might have an impact on loss to follow-up and viral load suppression (VLS). Although some studies revealed better outcomes on same-day ART initiation (SDI) [13-15], other studies have shown higher proportions of LTFU [16-20] and others did not show differences in LTFU comparing those on SDI and those who did not initiate ART on the same day (NSDI) [21-25]. While time to VLS was reduced among persons on SDI compared to those on NSDI [4], low proportion of VLS was documented among persons on SDI [26]. Similarly, like studies that assessed impact of SDI on LTFU, some showed superiority of SDI over NSDI on VLS [13,21,27,28], while others did not show superiority of SDI over NSDI on VLS [18,29].

The presence of mixed study findings of LTFU and VLS among persons living with HIV who initiated ART on the same day calls for the need to further evaluate its benefits. In addition, most of the prior evaluations included a small number of study participants. It has been seven years since WHO recommendations of test and treat were released, and over 90% HIV programs in LMICs have adopted these recommendations [30]. We anticipated many persons living with HIV were kept on SDI and through meta-analysis, a larger sample size can be obtained to improve on prior evaluations. The objective of this study was to provide pooled estimates on the proportion of LTFU and VLS and assess the effect of SDI on LTFU and VLS among people living with HIV in LMICs.

## Methods

**Registration:** this systematic review has been registered in the International Prospective Registry of Systematic Review with registration number CRD42023401767.

**Search strategy:** PubMed, Embase, Cochrane CENTRAL, clinicaltrials.gov and Google Scholar were searched between January 2016 and December 2022. Search terms were used to capture information on the SDI among people living with HIV in LMICs. Terms such as same-day ART initiation, rapid ART initiation, low- and middle-income countries, people living with HIV, and database specific MeSH were used. The search was restricted to papers written in English. No restriction was made on publication status.

**Selection criteria:** clinical trials and observational studies that involved people living with HIV in LMICs, reported relationship between SDI and LTFU or SDI and VLS and written in English were eligible for inclusion. We excluded studies that did not report the relationship between SDI and LTFU or VLS.

**Study selection and data abstraction:** the manuscripts searched from outlined databases were managed by Covidence software from which the final list of manuscripts was deduplicated. The National Institute for Health (NIH) tool was used to assess the quality of studies [31]. Reliability and validity of the measurement tools, participation rate, source of study participant recruitment, justification of sample size or power calculations, follow-up time, timing of exposure and outcome, proportion LTFU, potential confounders, appropriateness of statistical analysis were evaluated for all studies that met inclusion criteria. Finally, the quality of the studies was computed based on the stated parameters. Two review authors (HBM and HRO) independently completed the study selection for inclusion in the appraisal process. Disagreement between two independent reviewers for the inclusion of the manuscripts was handled by the third reviewer (MM). Using an Excel spreadsheet, two review authors (HBM and HRO) independently abstracted the following agreed upon data elements from the included studies: authors, year of publication, the country in which the study was conducted, study design, study period, timing of ART initiation, outcome, and sample size.

### Definition of variables

**Outcome:** the main outcome of interest was the proportion of people living with HIV who were LTFU. The proportion of LTFU was also categorized at 6 and 12 months of follow-up. Depending on the type of study, LTFU was defined as absence from clinic for more than 3-6 months from the expected date of drug pick-up. The secondary outcome was proportion of people living with HIV with virological suppression at 6, 12, and 24 months.

**Exposure:** the main exposure of interest was the timing of ART initiation, categorized as SDI for those who initiated ART on the same day of HIV diagnosis or NSDI for those who initiated ART on other days after HIV diagnosis.

**Statistical analysis:** we used forest plot to explore proportion of people living with HIV who were LTFU and those with VLS. Random effects models were used to account for study heterogeneity and an I^2^ statistic was used to test for heterogeneity. Finally, pooled proportions of LTFU and VLS were computed. In addition, the proportion of LTFU and VLS were also stratified by timing of ART initiation (SDI vs. NSDI) and type of study design (clinical trials vs observational studies). The effect of timing of ART initiation (SDI vs. NSDI) on LTFU and VLS were assessed using absolute risk differences (RDs) and corresponding 95% confidence intervals (CIs). The publication bias was assessed using funnel plot and the Egger regression test. For both heterogeneity and publication bias tests, a p-value <0.05 indicated the presence of heterogeneity and publication bias, respectively. To explore the source of heterogeneity, an influential analysis using the leave-one-out method was performed. Studies with missing information, such as those that reported proportion of LTFU or VLS without actual numerators and/or denominators, were excluded from the analysis. Summaries of study characteristics and associations between timing of ART initiation and LTFU or VLS were tabulated. All statistical tests were performed using Stata version 18 (Stata Corporation, College Station, Texas, USA).

## Results

**Figure 1:**
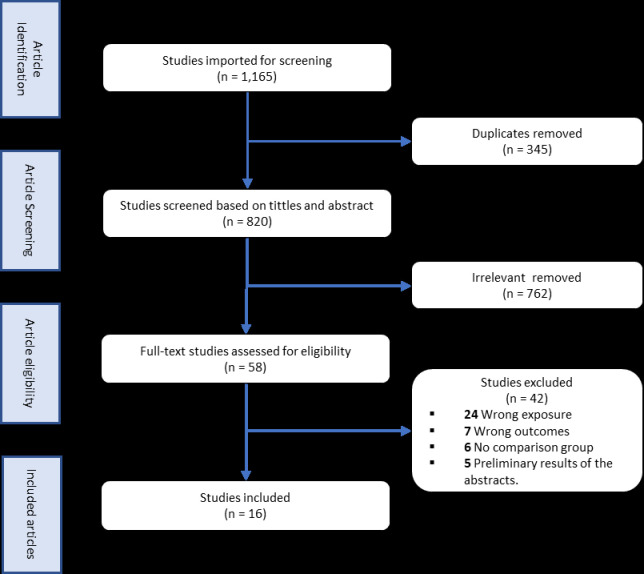
PRISMA flow diagram of the included studies for meta-analysis of same day ART initiation among persons living with HIV in low- and middle-income countries

A total of 1,165 articles were retrieved through searches, of which 345 were duplicates and removed. Titles and abstracts were screened for 820 articles, of which 762 were excluded. The remaining 58 received a full review, of which 16 were eligible for final analysis (Figure 1). One study met eligibility criteria but was excluded because numerator and denominator of the outcome of interest were missing [32].

**Characteristics of studies included:** study designs for articles included clinical trials (n = 5) [13,21,24,27,28], cohorts (n = 10) [16-20,22,23,25,26,29] and cross-sectional studies (n = 1) [15]. The sample size for studies ranged from 274 to 92,609. A total of 171,111 people living with HIV were included in this analysis. We included studies from Africa (n = 13), Asia (n = 1), and Latin America (n = 2) (Table 1).

**Table 1:** summary of characteristics of the included studies

Publication	Country	Study design	year study done	Sample size	VLS			LTFU			Quality scores
					Time point	SDI	NSDI	Time point	SDI	NSDI	
Ross *et al*. 2022	multi country data	cohort	2015-2018	29017	6-month	88.1	88.1	12-month	27.7	20.6	77.0
Kerschberger *et al*. 2021	Eswatini	cohort	2014-2016	1328				-	31.6	21.2	77.0
Lilian RR *et al*. 2020	South Africa	cohort	2017-2018	32290				6-month	29.2	20.7	77.0
Mshweshwe *et al*. 2020	South Africa	cohort	2017	826	6-month	78.1	80.3				92.0
Davey *et al*. 2020	South Africa	cohort	2016-2018	92609				-	33.3	31.9	85.0
Ahmed *et al*. 2021	Ethiopia	cohort		877	12-month	73.4	83.7				92.0
Amstutsi *et al*. 2020	Lesotho	Clinical trial	2016	274	24-month	56.9	54.0				70.0
Koenig *et al*. 2017	Haiti	Clinical Trial	2015	703	12-month	61.1	51.7	-	17.3	22.5	75.0
Mitiku *et al*. 2016	Ethiopia	cohort	2015	343				-	23.1	11.7	77.0
Seekaew *et al*. 2020	Thailand	cohort	2017-2019	648				12-month	4.2	5.8	85.0
Labhardt *et al*. 2018	Lesotho	Clinical trial	2016	274	12-month	50.4	34.3	6-month	8.8	7.3	100.0
Dorvil N *et al*. 2021	Haiti	Clinical trial	2017-2019	500	24-month	62	68.4				92.0
Onoya *et al*. 2021	South Africa	cohort	2015	410				12-month	47.7	47.2	85.0
Kimanga *et al*. 2022	Kenya	Cohort	2015-2018	8592	6-month	85.9	86.1				90.0
Barnabas *et al*. 2020	South Africa & Uganda	Clinical Trial	2016-2019	1315	12-month	73.9	63.1				77.0
Msongole *et al*. 2019	Tanzania	Cross-sectional	2018	1105	12-month	87.9	83.9				83.0

* SDI, same day ART initiation; NSDI, non-same day ART initiation; RD, risk difference; LTFU, lost to follow up; VLS, viral load suppression

**Pooled prevalence of LFTU and subgroup analysis:** overall, nine articles with 157,622 people living with HIV were analyzed for LTFU. The overall pooled proportion of LTFU was 22.0% (95% CI: 18.5-25.7%). Subgroup analysis showed that the pooled prevalence of LTFU for cohort studies was 24.4% (95% CI: 20.5-28.6%) and from clinical trials was 16.1% (13.9- 18.5%) (Table 2).

**Table 2:** loss to follow and viral load suppression among persons living with HIV in low- and middle-income countries: comparison between same-day and non-same-day ART initiations

Indicator	# Study	SDI		NSDI		Absolute RD, (95% CI)	I^2^	
		# PLWH	Pooled estimate % (95% CI)	# PLWH	Pooled estimate % (95% CI)			
**Viral load suppression**								
Overall	10	11,694	74.0 (66.7 - 80.6)	11,126	71.9 (63.8 - 79.3)	0.02 (-0.03 - 0.07)	92.4	
Duration	-	-	-	-	-	-	-	
6-month	3	9,611	85.7 (82.4 - 88.6)	8,693	85.9 (83.2 - 88.4)	-0.00 (-0.01 - 0.01)	0.02	
12-month	5	1,727	70.4 (58.1 - 81.4)	2,072	64.7 (47.2 - 80.4)	0.05 (-0.03 - 0.14)	89.9	
24-month	2	356	65.6 (60.6 - 70.5)	361	68.3 (63.4 - 73.0)	-0.02 (-0.10 - 0.06)	25.6	
Study design	-	-	-	-	-	-	-	
Clinical trial	5	1,254	63.2 (54.6 - 71.4)	1,280	56.3 (43.9 - 68.4)	0.07 (-0.01 - 0.14)	71.5	
Cohort/Cross-sectional	5	10,440	83.5 (79.2 - 87.5)	9,846	85.2 (83.0 - 87.2)	-0.01 (-0.06 - 0.03)	91.5	
**LTFU**								
Overall	9	66,841	23.8 (20.7 - 27.0)	90,289	20.9 (16.2 - 26.1)	0.04 (0.01 - 0.07)	96.4	
Duration	-	-	-	-	-	-	-	
6-month	2	13,175	28.8 (28.1 - 29.6)	19,389	20.6 (20.0 - 21.1)	0.06 (-0.01 - 0.12)	68.7	
12-month	3	18,934	23.4 (6.0 - 47.5)	10,629	22.9 (7.4 - 43.4)	0.03 (-0.03 - 0.10)	77.1	
Study design	-	-	-	-	-	-	-	
Clinical trial	2	484	14.6 (11.6 - 17.9)	493	17.6 (14.3 - 21.1)	-0.02 (-0.09 - 0.05)	55.1	
Cohort/Cross-sectional	7	66,357	26.7 (23.3 - 30.1)	89,796	22.9 (17.4 - 28.9)	0.05 (0.02 - 0.08)	95.7	

* SDI, same day ART initiation; NSDI, non-same day ART initiation; RD, risk difference; LTFU, lost to follow up; VLS, viral load suppression

**SDI and its association with LTFU:** compared to those who received NSDI, SDI had a significant increased risk of LTFU (RD = 0.04; 95%CI: 0.01-0.07) (Table 2). Among the nine studies included, only five reported 6- and 12-month LTFU, and four did not specify the time point for LTFU. For these five studies, the pooled proportion of LTFU was 24.0% (95% CI: 23.5-24.4%) and 22.9% (95% CI: 7.2-44.0%) at 6 and 12 months, respectively. There was an increased risk of LTFU among persons who received SDI compared to NSDI, although not statistically significant, at 6 months (RD = 0.06, 95% CI: -0.01-0.12) and 12 months (RD = 0.03, 95% CI: -0.03-0.10). Observational studies showed increased risk of LTFU among persons who received SDI compared to NSDI (RD = 0.05, 95% CI: 0.02-0.08), however, clinical trials did not show a statistically significant difference (RD = -0.02, 95% CI: -0.09-0.05).

**Figure 2:**
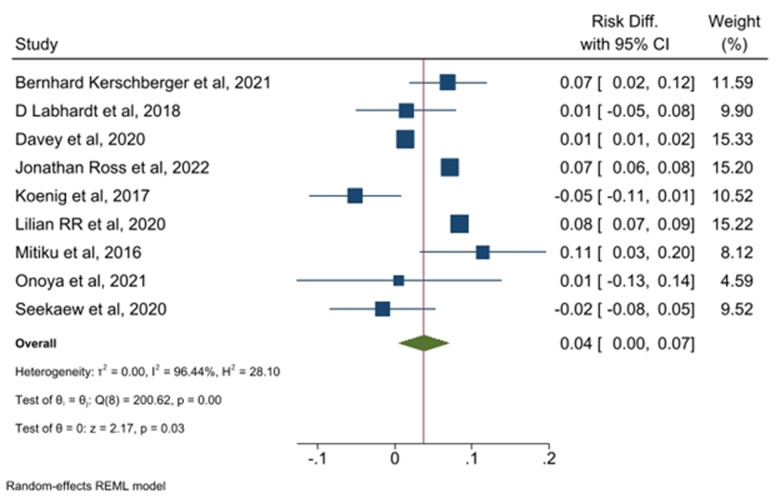
loss to follow-up among persons living with HIV in low- and middle-income countries: comparison between same-day ART initiations and non-same-day ART initiation

**Figure 3:**
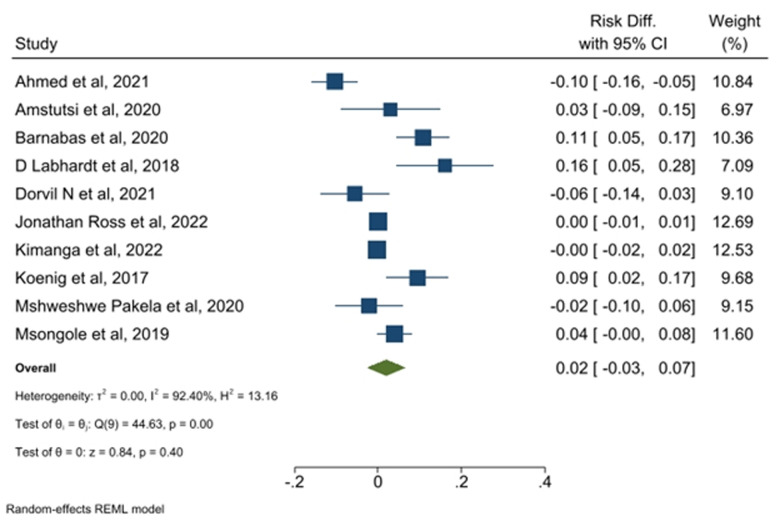
viral load suppression among persons living with HIV in low- and middle-income countries: comparison between same-day ART initiations and non-same-day ART initiation

**Figure 4:**
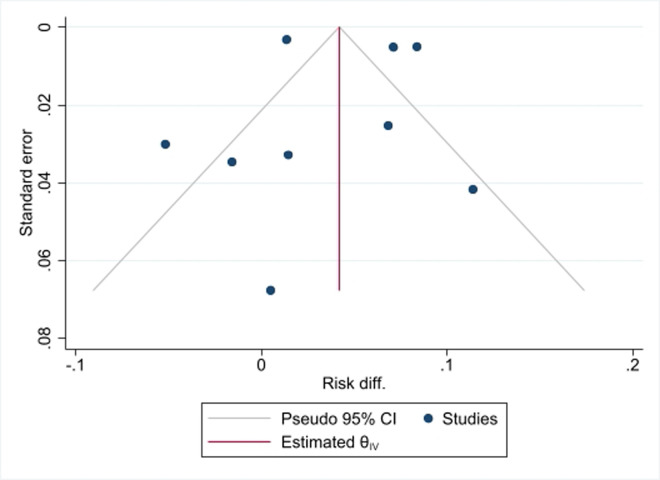
funnel plot for publication bias on studies that assessed impact of same-day ART initiation on lost to follow-up, Egger's regression test (p = 0.4392)

**Figure 5:**
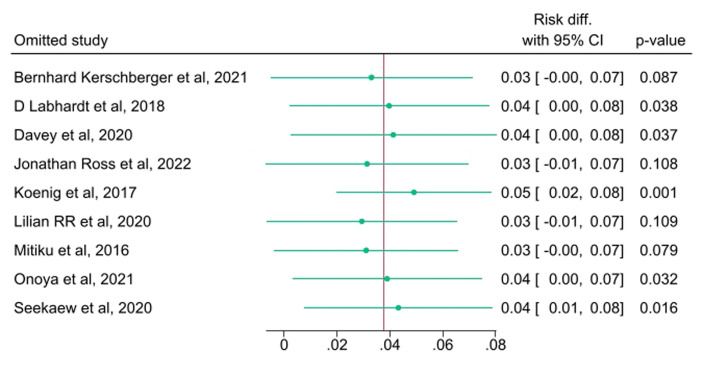
inferential analysis exploring sources of heterogeneity on studies that assessed impact of same-day ART initiation on lost to follow-up

**Assessment of heterogeneity, publication bias, and influential analysis on LTFU:** the I^2^ statistic test of heterogeneity showed a Q value of 200.62 (p<0.001) on the risk difference of LTFU among SDI vs NSDI (Figure 2, Figure 3). As shown in Figure 4, the funnel plot had few data points outside the funnel and the Egger's test showed no statistically significant small-study effect (z-test = -0.77, p=0.4392), indicating absence of publication bias. Estimates from the leave-one-out forest plot showed a significant influence of some studies on the risk difference of LTFU comparing those with SDI vs NSDI (Figure 5).

**Pooled prevalence of VLS and subgroup analysis:** ten articles with 43,483 people living with HIV were analyzed for VLS and the pooled prevalence of VLS was 72.7% (95% CI; 65.4-79.5%). When stratified by study design, the pooled proportion of VLS was 84.1% (95% CI: 81.3-86.8%) for observational studies and 59.6% (95% CI: 49.6-69.2%) for clinical trials.

**SDI and its association with VLS:** there was no statistically significant difference in VLS comparing those who received SDI to NSDI (RD = 0.02, 95% CI: -0.03-0.07) (Table 2). Among the 10 studies included, three reported 6- month VLS, five reported 12-month VLS, and two reported 24-month VLS. The overall 6-, 12-, and 24-month VLS was 85.4% (95% CI: 82.7-88.0%), 67.4% (95% CI: 53.0-80.2%), and 66.9% (95% CI: 63.4-70.3%), respectively. There was no statistically significant difference in VLS among those who had SDI compared to NSDI at 6 months (RD = -0.00, 95% CI: -0.01-0.01), 12 months (RD = 0.05, 95% CI: -0.03-0.14), and 24 months (RD = -0.02, 95% CI: -0.10-0.06). There was no statistically significant difference in VLS among those who had SDI compared to NSDI for observational studies (RD = -0.01, 95% CI: -0.06-0.03) and for clinical trials (RD = 0.07, 95% CI: 0.01-0.14).

**Figure 6:**
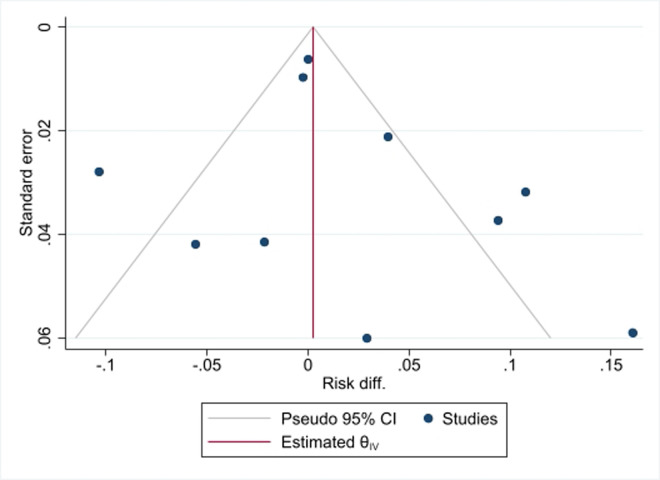
funnel plot for publication bias on studies that assessed impact of same-day ART initiation on viral load suppression, Egger's regression test (p = 0.3774)

**Figure 7:**
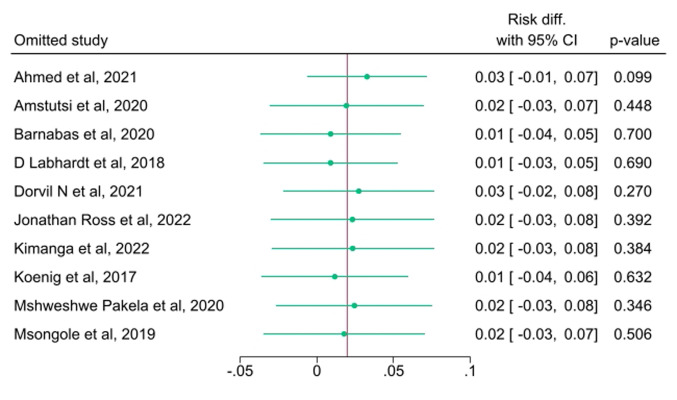
inferential analysis exploring sources of heterogeneity on studies that assessed impact of same-day ART initiation on viral load suppression

**Assessment of heterogeneity, publication bias, and influential analysis on VLS:** the I^2^ statistic test of heterogeneity showed a Q value of 44.63 (p<0.001) on the risk difference for VLS among SDI vs NSDI (Figure 3). Four of the studies fell outside the funnel as shown on the funnel plot (Figure 6). There was no significant small-study effect based on the Egger's test statistic (z-test = 0.93, p=0.3774). As shown on the leave-one-out forest plot (Figure 7), the risk difference was not significantly driven (p>0.05) by a single study evaluated SDI vs NSDI on VLS.

## Discussion

We conducted a systematic review and meta-analysis among people living with HIV in LMICs to evaluate pooled estimates of LTFU and VLS, as well as assessing the effect of SDI on LTFU and VLS. The overall pooled estimates of LTFU and VLS in these settings were 22% and 73%, respectively. Overall, the risk of LTFU was 4% higher among participants who initiated ART on the same day compared to those who did not initiate treatment on the same day. Although there was an increased risk of LTFU among persons living with HIV who initiated treatment on the same day compared to those who did not initiate treatment on the same day, VLS between these two groups were comparable.

Even after several years of advocating the benefits of remaining in care among people living with HIV, an appreciable number of people is still lost to follow-up. Because HIV treatment is a life-long process and interrupted treatment is associated with poor patient outcomes and a higher risk of ongoing HIV transmission and development of drug resistance [33,34], interventions that support and keep patients on continued care are critical. The concept of SDI is intuitive in that it reduces time to VLS and, overall, reduces community viral load and chances of HIV transmission. Prior data showed that patients out of care account for 60% of new HIV infections [35]. These data underscore the need to retain people living with HIV in care to control the HIV epidemic. The increased risk of LTFU among persons who received SDI requires additional interventions to ensure retention in care. HIV-associated stigma, disclosure, time to process and accept an HIV diagnosis, and time needed to decide to commit to a lifelong treatment are some of the barriers for SDI identified from previous qualitative studies [36-38]. Since different people have different barriers, assessment, and identification of the potential barriers of ART initiation and retention, and implementation of interventions targeted to the identified barriers, would reduce LTFU. Barrier analysis intervention has been shown to improve retention among people living with HIV in Kenya [39]. Although other structural and healthcare facility factors contribute to LTFU, HIV-related stigma remains a predominant concern among people living with HIV [37]. If these concerns are adequately addressed, potential benefits of SDI may be maximized.

We did not find a statistically significant difference in VLS among individuals who initiated ART treatment on the same day compared to those who initiated beyond the first day of HIV diagnosis. This could be due to the fact that persons who have established treatment, and are taking their medication as prescribed, are expected to achieve VLS within 6 months of treatment [40]. In this analysis, we reported VLS at 6, 12 and 24 months; at these time points, differences in VLS could be attributable to some other factors such as non-adherence in addition to timing of ART initiation. SDI is likely to have an immediate impact on shorter-term outcomes, such as retention, than on long-term outcomes, such as VLS. Although pooled estimates of VLS were comparable between persons on SDI and NSDI, reviewed literature showed benefits of SDI. For example, time to VLS is shorter among persons on SDI compared to those on NSDI [4]. The median time to VLS among persons on SDI was 56 days compared to 79 days for those on NSDI. Median time to VLS among rapid starters (those who initiated treatment within 7 days) was 55 days compared to 77 days among non-rapid starters (those who initiated treatment beyond 7 days) [41]. These data support the need to rapidly initiate treatment for people living with HIV, particularly on the same day of HIV diagnosis. Furthermore, since it is also possible that HIV testing be done at a site that does not offer HIV treatment, this may be a barrier to SDI. In these circumstances, improving referral systems and follow-through is paramount to ensure newly diagnosed persons are immediately initiated on treatment.

This evaluation is not without limitations. Although the concept of LTFU is the same, this systematic review involved studies with varying definitions of LTFU. Using a few days of absence from the last clinic visit may overestimate the prevalence of LTFU and using many days of absence from the last clinic visit may underestimate it. Some studies compared SDI against those who initiated ART between one- and 30-days post-HIV diagnosis, others included those who initiated treatment beyond 30 days. This variation in the comparison group might have underestimated the prevalence of LTFU. Persons who had enough time to accept their HIV diagnosis prior to ART initiation may be more likely to remain in care. Furthermore, because the search was limited to publications written in English, it is likely that other relevant publications from non-English journals were missed. The main strength of this research is the inclusion of many studies with pooled estimates from a large sample size. This meta-analysis remains relevant as it provides estimates of LTFU and VLS and how these outcomes are impacted by SDI among persons living with HIV in LMICs.

## Conclusion

Despite known benefits of ART among people living with HIV, LTFU remains a threat to achieving maximal benefits. In this analysis, nearly two in ten people living with HIV in LMICs who initiated ART were LTFU. SDI was associated with increased risk of LTFU; therefore, further exploration to determine reasons for LTFU is critical. Healthcare professionals providing services to people living with HIV should closely monitor patients who initiate ART on the same day of HIV diagnosis to minimize chance of LTFU. Identifying reasons for LTFU and implementing strategies to retain people in care are particularly important as this meta-analysis showed comparable long-term viral load suppression between those who initiate treatment on the same day of HIV diagnosis versus those who initiate later.

### What is known about this topic

- *The World Health Organization recommended early antiretroviral therapy (ART) initiation after HIV diagnosis because early ART initiation reduces time to viral load suppression and subsequently HIV transmission;*
- *Pretreatment counselling sessions are important in ensuring people living with HIV get time to understand and accept HIV diagnosis;*
- *There has been conflicting evidence about loss to follow-up and viral load suppression among those who initiate treatment on the same day compared to those who initiate treatment beyond the same day of HIV diagnosis*.

### What this study adds

- *This study remains relevant as it provides pooled estimates of loss to follow-up and viral load suppression and how these outcomes are impacted by same-day ART initiation among persons living with HIV in low- and middle-income countries;*
- *This study provides signals to healthcare workers in providing close follow-up for persons receiving ART on the same day of HIV diagnosis to ensure maximum retention in care;*
- *This study emphasizes that same-day ART initiation is not inferior to initiation of ART beyond the same day of HIV diagnosis in terms of viral load suppression*.
